# Theoretical analysis of the mechanisms of a gender differentiation in the propensity for orthostatic intolerance after spaceflight

**DOI:** 10.1186/1742-4682-7-8

**Published:** 2010-03-18

**Authors:** Richard L Summers, Steven Platts, Jerry G Myers, Thomas G Coleman

**Affiliations:** 1 Department of Emergency Medicine, University of Mississippi Medical Center, Jackson, Mississippi 39216, USA; 2 Cardiovascular Laboratory, Space Life Sciences Directorate, National Aeronautics and Space Administration Johnson Space Center, Houston, Texas 77058, USA; 3 Human Research Office, NASA Glenn Research Center, Cleveland, Ohio 44135, USA

## Abstract

**Background:**

A tendency to develop reentry orthostasis after a prolonged exposure to microgravity is a common problem among astronauts. The problem is 5 times more prevalent in female astronauts as compared to their male counterparts. The mechanisms responsible for this gender differentiation are poorly understood despite many detailed and complex investigations directed toward an analysis of the physiologic control systems involved.

**Methods:**

In this study, a series of computer simulation studies using a mathematical model of cardiovascular functioning were performed to examine the proposed hypothesis that this phenomenon could be explained by basic physical forces acting through the simple common anatomic differences between men and women. In the computer simulations, the circulatory components and hydrostatic gradients of the model were allowed to adapt to the physical constraints of microgravity. After a simulated period of one month, the model was returned to the conditions of earth's gravity and the standard postflight tilt test protocol was performed while the model output depicting the typical vital signs was monitored.

**Conclusions:**

The analysis demonstrated that a 15% lowering of the longitudinal center of gravity in the anatomic structure of the model was all that was necessary to prevent the physiologic compensatory mechanisms from overcoming the propensity for reentry orthostasis leading to syncope.

## Background

The microgravity environment provides an intriguing new laboratory in which we can develop a deeper understanding of the relationship between the functioning of human physiology and the structural anatomic platform within which it performs. In this paper we hypothesize that a simple common anatomic difference between males and females can have a substantial impact on their respective abilities to respond to a transitioning between the space and earth environments. Astronauts returning from spaceflight are frequently found to have orthostatic intolerance (OI) upon reentry into the earth environment [1]. Women have been found to have a much greater predisposition to the development of OI postflight than their male counterparts under similar circumstances [2,3]. In the past, these differences have been attributed to very complex physiologic mechanisms involving endocrine, neurologic and cardiac components of circulatory control [1,4-7]. However, a simpler explanation might be achieved by looking at some basic anatomic differences between men and women.

It is well know that on average women commonly have an 8 - 15% lower longitudinal center of gravity (COG or center of mass relative to height) than men [8-10]. This anatomic differentiation has been speculated to have arisen evolutionarily during the development of bipedal locomotion as a means to provide better stability in females during pregnancy and infant carriage [11]. The differences also impact inherent athletic agility and are a major contributing factor to techniques used in sporting events [8,9,12]. In gymnastics, women excel in uneven bar competitions while men usually compete on parallel bar configurations.

Moment to moment control of blood pressure is determined by an integration of physiologic mechanisms coordinating the maintenance of blood flow through a balance of both preload and afterload [13]. Arterial perfusion pressure is dependent upon the peripheral resistance and cardiac output. The preload determines the venous return which in turn drives the cardiac output through the Starling mechanism. In a previous computer analysis, we postulated that the typical loss of extracellular fluid volume during spaceflight reduced the external compressive forces produced by the interstitial fluid pressure on the capacitance veins of the lower extremities and resulted in a reduced venous compliance and a sequestration of blood when the subject was made to stand upright [14]. The sequestration of blood in the lower extremities diminished the preload and venous return and appears to be the initiating factor in the physiologic cascade to developing OI postflight. A similar type of mechanism has subsequently been proposed by other researchers using computer models [15]. Emerging experimental evidence from spaceflight analog studies appears to also support this hypothesis [16,17]. Compensatory mechanisms such as neurohormonal counterregulatory systems can overcome this driving force of OI by increasing the venous tone. A failure of these counterregulatory systems might be contributing to a predisposition of OI in some returning astronauts [18]. However, it is not clear why women as a group should be so overwhelmingly predisposed.

## The Proposed Hypothesis

Since a larger portion of the body mass of women is situated in the lower tissues of the legs and pelvis, it could also be assumed that there is a relatively larger interstitial space in this area. As the interstitial spaces become dehydrated during spaceflight, a proportionately greater fluid volume is required to refill these spaces in women as compared to men upon return to earth. These differences at the time of reentry could result in relatively less external compression on the lower extremity venous capacitance vessels by fluid within the interstitial spaces and a greater percent of sequestered blood for women as compared to men. If a lower center of gravity for women is considered to be the primary differentiating factor involved in their greater susceptibility to orthostatic stress after spaceflight through this proposed mechanism, we should see a failure in the overall system to maintain venous return upon standing and a greater propensity for the OI to develop with this isolated anatomic perturbation. To examine the theoretical consistency of this hypothesis, we employed the use of a complex computer model of human physiology that incorporates many of the relevant mechanisms considered in circulatory control [14,19,20]. The model will allow us to perform computer simulation studies of orthostatic stress on two identical physiologic systems with only a variance in their longitudinal center of gravity.

## Computer Systems Analysis of the Hypothesis

The hypothesis was examined through a systems analysis approach using a derivative of a well-established computer model of circulatory functioning previously described in the literature [14,19,20]. This methodology allows us to develop a sophisticated approach to hypothesis formulation and a detailed analysis of this very complex physiologic process [21]. Such a technique has been used successfully to understand mechanisms pertaining to hypertension, fluid volume control and myocardial structural changes that were not intuitively obvious otherwise [22].

The computer model contains over 4000 parameters that describe the detailed interaction of multiple aspects of the circulatory system as determined by basic hydraulics as well as neural, endocrine and metabolic control mechanisms. A compiled working version of the model is available for download and can be found at http://physiology.umc.edu/themodelingworkshop. The integrated relationships are based upon basic physical principles and well-established functional physiologic interactions that incorporate the physical responses to changes in pressures, flows and hydraulics as affected by gravitational forces. Other specifics peculiar to an exposure to microgravity including baroreceptor deconditioning in response to chronic intravascular pressure and volume changes, compartmental fluid shifts as well as the special anatomic differences in men and women were also incorporated into the structure of the mathematical model. Since much of the past research on the postflight OI issue has focused on the complex interactions of autonomic, neurohormonal and cardiac elements, this large model incorporates most of these factors. These physiologic systems are allowed to adapt in a manner that has been demonstrated experimentally (20). The female gender differences were simulated by a 15% caudal shift in the longitudinal center of gravity of the model's anatomic structure with a resulting relative increase in the proportional mass in the lower body compartment. However, no additional gender distinctions were otherwise incorporated in the physiologic functioning of the model. The newly formulated model was then used in a series of computer simulation studies solved on a Windows-based PC using standard numerical methods to examine the proposed hypothesis. The analytic procedure involves recreating the experimental protocol of exposure of a virtual astronaut to spaceflight, returning to earth and subsequent tilt testing in 1-G for a virtual astronaut in a computer simulation environment. In the computer study, the physiologic interactions of the model were allowed to adjust to the conditions of spaceflight by removal of the effects of gravity on hydraulics from the model components for a simulated period of one month (see appendix). These gravitational effects were returned to the model at the point of reentry into the earth environment. Differences in orthostatic tendency in the male and female virtual astronauts were then compared immediately upon return to earth when they were force to assume an upright posture in the simulations.

## Results

**Figure 1:**
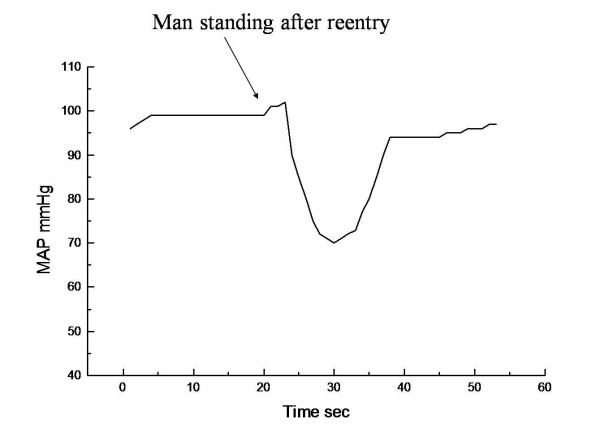
**The computer simulation study demonstrate the orthostatic mean arterial blood pressure (MAP) response in man when assuming upright posture upon reentry to earth's gravity after one month in the space environment**. The drop in blood pressure is quickly corrected by compensatory physiologic regulatory mechanisms.

**Figure 2:**
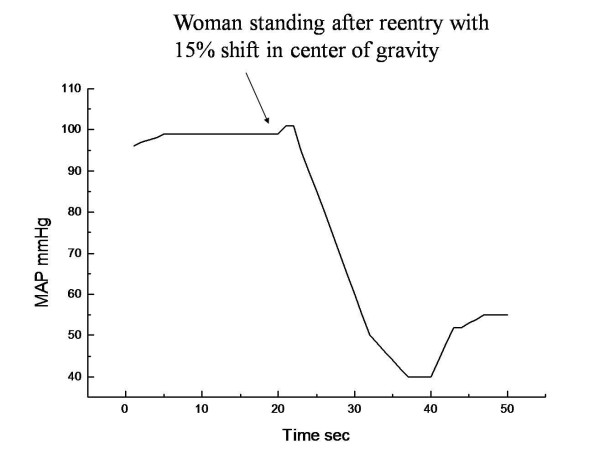
**The second computer simulation study using the same model with only a change in the center of gravity (woman model) demonstrates a similar orthostatic blood pressure response when assuming upright posture after reentry but with a failure of the compensatory physiologic responses to correct the hypotension**.

The results of the simulations studies are displayed as graphical outputs in figures 1, 2. The utility of the model to replicate the postflight orthostatic condition in a man returning from spaceflight was validated in the simulation study (figure 1). The typical drop in mean arterial pressure upon standing after reentry is followed rapidly by a correction of the pressure to near normal values due to the compensatory physiologic response mechanisms (i.e. baroreceptors, etc.). In the second simulation study, the same exact physiologic model was used as in the first simulation with the exception of a change in the anatomic distribution of mass with a 15% lower longitudinal center of gravity to simulate the structure of a woman. The graphical representation of the model output (figure 2) reveals that unlike the prior simulation of a man, the simulation of the woman upon reentry is unable to compensate for the factor initiating the initial drop in pressure. Advanced analysis of the simulation history reveals that there is a proportionately greater sequestration of blood in the capacitance veins of the lower extremities of the woman during the movement to the upright posture due to effective differences in vascular compliances as compared to the man. The resultant limitation on venous return is translated into a more profound drop in MAP without sufficient compensatory response as is commonly seen in conditions of OI. Since we did not differentiate the male model from the female model except for the change in COG than these physiologic events were the only significant differences expected.

## Discussion

Reentry orthostasis secondary to a prolonged exposure to microgravity has been a common problem for returning astronauts since the beginning of manned space exploration. The mechanisms involved have been poorly understood due to the many physiologic control systems involved. Recently, a detailed systems analysis approach to the problem was used to integrate many of the experimental findings in to a more comprehensive theory of understanding [14]. The analysis suggested that changes in the capacitance of the lower extremity veins secondary to a loss of external fluid forces in the dehydrated extracellular tissue compartment was associated with a propensity for reentry orthostasis. However, still lacking is an insight into the reasons for a greater predisposition of orthostatic intolerance among women astronauts. In the current paper we hypothesize that potential for OI is accentuated in women due to their inherent lower center of gravity and proportionately larger mass in the lower extremities. Examining the theoretical consistency of the hypothesis in the context of the current considered mechanism for OI induction suggests that a change in the COG of the individual, without other gender differentiating physiologic etiologies, was all that was necessary to increase the likelihood for the development of OI postflight.

The major premise of the hypothesis is centered around the concept that the interstitial spaces of the lower extremities of women are proportionately larger than that for men of the same size. As fluid become mobilized and shifts cephalad and away from these spaces upon exposure to microgravity, this creates a relative sink that must be refilled once the individual returns to earth. The relative dehydrated state of these spaces during spaceflight has a two-fold effect to potentiate a condition of OI in returning astronauts. First, the gravitational forces that relocating blood back into the lower body upon return to earth result in a transudation of fluid into the interstitium of the legs and removes fluid from an already contracted plasma volume. Secondly, the reduced fluid pressure within these dehydrated lower extremity interstitial spaces produces less external compression on the venous capacitance vessels and results in a sequestration of blood volume in the lower body. Both of these factors work to reduce the preload to attenuate venous return. If the lower extremity interstitial spaces of women are larger than for men, then we would see an amplification of this effect.

There is limited information in the literature describing the gender differences in physiologic responses surrounding the orthostatic event. However, it does appear that women are generally more susceptible to reflex syncope in common clinical situations [23]. There is evidence to suggest that net capillary fluid filtration is greater in women as compared to men during application of lower body negative pressure [24]. There is also data to suggest that the OI propensity in women is primarily due to a reduced ability to maintain venous return and cardiac filling rather than a diminished responsiveness of vascular resistance [2,25,26]. These studies support the proposed hypothesis and are consistent with our general understanding of the development of OI.

It is important to note that all returning women astronauts do not have OI and there are still a significant percent of male astronauts who do develop OI. This observation implies that gender based physiologic mechanisms that are absolute (i.e. sex hormone differences) are unlikely to be considered as a definitive answer to the OI differentiation. While our hypothesis embraces a higher propensity for women to develop OI postflight due to a likelihood of a lower COG, the concept is developed in the context that any individual of either sex could experience orthostasis based upon the particulars of their circulatory system. Though it is clear that women in general tend to have a lower COG, there is some variability in the distribution of the COG within the overall population (male and female) and some women may have a higher COG while some men have a lower COG [10]. The graphs in the results are intended to reflect the propensity of females to have OI as a result of their population average of a lower COG. A finding of a lower COG and a resultant postflight OI tendency might not be necessarily true for any individual astronaut (male or female). The study was intended to propose a mechanism explaining why the female population as a whole has a higher propensity for OI. An analysis of OI tendencies postflight in association with each subject's COG (male and female) would be an important test of this hypothesis.

## Competing interests

The authors declare that they have no competing interests.

## Authors' contributions

RLS is the main author and developer of the theoretical concept presented. SP provided collaborative input based upon his expertise and possession of correlative data. JGM provided computational expertise and theoretical review of the paper contents. TGC is the primary model developer. All authors have read and approved the final manuscript.

## Appendix

### Steps in the simulation

#### Man

Load the executable form of the model

Turn the daily planner option under the "clock" icon to off

Run the model under the "Go" menu option for 12 hours to stabilize

Under the 'position" icon move the gravity slidebar to the 0 position

Run the model under the "Go" menu option for 1 month

Under the 'position" icon move the gravity slidebar to the 1 position

Under the same icon set the "restraint" option to tilt

Move the tilt slidebar to 90 degrees

Run the model under the "Go" menu option for 1 minute

#### Woman

Load the executable form of the model

Turn the daily planner option under the "clock" icon to off

Run the model under the "Go" menu option for 12 hours to stabilize

Under the "IFV" icon go to the lower torso setting

Set the normal volume slidebar in the interstitial space panel to 15% higher volume

Under the "IFV" icon go to the upper torso setting

Set the normal volume slidebar in the interstitial space panel to 15% lower volume

Under the 'position" icon move the gravity slidebar to the 0 position

Run the model under the "Go" menu option for 1 month

Under the 'position" icon move the gravity slidebar to the 1 position

Under the same icon set the "restraint" option to tilt

Move the tilt slidebar to 90 degrees

Run the model under the "Go" menu option for 1 minute
